# Influenza A virus NS1 optimises virus infectivity by enhancing genome packaging in a dsRNA-binding dependent manner

**DOI:** 10.1186/s12985-020-01357-3

**Published:** 2020-07-16

**Authors:** Tim Wai Sha, Michaela Weber, Dacquin M. Kasumba, Takeshi Noda, Masahiro Nakano, Hiroki Kato, Takashi Fujita

**Affiliations:** 1 Laboratory of Molecular Genetics, Institute for Frontier Life and Medical Science, Kyoto University, Kyoto, Japan; 2 Laboratory of Molecular and Cellular Immunology, Graduate School of Biostudies, Kyoto University, Kyoto, Japan; 3 Laboratory of Ultrastructural Virology, Graduate School of Medicine, Kyoto University, 53 Shogoin Kawahara-cho, Sakyo-ku, Kyoto, 606-8507 Japan; 4 Laboratory of Ultrastructural Virology, Institute for Frontier Life and Medical Sciences, Kyoto University, 53 Shogoin Kawahara-cho, Sakyo-ku, Kyoto, 606-8507 Japan; 5 Institute of Cardiovascular Immunology, University Hospital Bonn, University of Bonn, Bonn, Germany

**Keywords:** Influenza a virus, Non-structural protein 1, Innate immunity, Genome packaging, Virus-like production system, dsRNA binding domain

## Abstract

**Background:**

The non-structural protein 1 (NS1) of influenza A virus (IAV) is a key player in inhibiting antiviral response in host cells, thereby facilitating its replication. However, other roles of NS1, which are independent of antagonising host cells’ antiviral response, are less characterised.

**Methods:**

To investigate these unidentified roles, we used a recombinant virus, which lacks NS1 expression, and observed its phenotypes during the infection of antiviral defective cells (RIG-I KO cells) in the presence or absence of exogeneous NS1. Moreover, we used virus-like particle (VLP) production system to further support our findings.

**Results:**

Our experiments demonstrated that IAV deficient in NS1 replicates less efficiently than wild-type IAV in RIG-I KO cells and this replication defect was complemented by ectopic expression of NS1. As suggested previously, NS1 is incorporated in the virion and participates in the regulation of viral transcription and translation. Using the VLP production system, in which minigenome transcription or viral protein production was unaffected by NS1, we demonstrated that NS1 facilitates viral genome packaging into VLP, leading to efficient minigenome transfer by VLP. Furthermore, the incorporation of NS1 and the minigenome into VLP were impaired by introducing a point mutation (R38A) in the double stranded RNA-binding domain of NS1.

**Conclusion:**

These results suggest a novel function of NS1 in improving genome packaging in a dsRNA binding-dependent manner. Taken together, NS1 acts as an essential pro-viral regulator, not only by antagonizing host immunity but also by facilitating viral replication and genome packaging.

## Background

Influenza A virus (IAV) segment 8 encodes two proteins known as non-structural protein 1 (NS1) and nuclear export protein (NEP). The NS1 protein has been well recognised as a counteractor of host cell innate immunity [1–9]. Both in vitro and structural studies suggested that NS1 inhibits host cell antiviral response by directly targeting RIG-I, which is the pattern-recognition receptor (PRR) for inducing type-I interferon (IFN-I) upon IAV infection [10–12]. It has been also suggested that NS1 targets the E3 ligase tripartite motif- containing protein 25 (TRIM25) and activator of the IFN-induced protein kinase (PACT), which prevents activation of RIG-I and induction of IFN-I [13–15]. Taken together, NS1 negatively regulates RIG-I-dependent host cell antiviral responses through a board range of mechanisms.

Although the role of NS1 in antagonising the host cell antiviral response is well characterised, its role in the viral life cycle is less clarified. Several studies found that NS1 regulates viral mRNA transcription and translation [16–21], which are the replication steps in the viral life cycle. Of note, a recent study reported that a low amount of NS1 was detected in purified virus particles [22], suggesting that NS1 is pre-packaged within the virus particles during virus assembly. Moreover, a dsRNA-binding protein STAUFEN was reported to be packaged within the human immunodeficiency virus (HIV) particles and its role in supporting HIV genome packaging was also demonstrated [23]. Considering that NS1 is also a dsRNA-binding protein and pre-packaged in IAV particles, these reports led us to re-examine the potential function of NS1 within the viral life cycle of IAV.

We examined the effects of exogenously expressed NS1 in RIG-I knock out (RIG-I KO) cells infected with recombinant IAV, which lacks NS1 gene expression. Furthermore, we employed a virus-like particle (VLP) production system to investigate the involvement of NS1 in the viral life cycle. Our findings suggest that NS1 supports efficient virus production and viral genome packaging to maximize IAV infectivity, which are independent of antagonistic effects on RIG-I antiviral signalling.

## Methods

### Cells and viruses

MDCK [24] and 293 T RIG-I KO [25] were cultivated in Dulbecco’s modified Eagle’s medium (DMEM) (Nacalai Tesque) supplemented with 5% foetal calf serum (FCS) (Gibco) and 1% penicillin and streptomycin (Nacalai Tesque) at 37 °C and 5% CO_2_. A/Puerto Rico/8 (H1N1) (PR8-WT) and recombinant virus deficient in the NS1 gene (PR8-delNS1) [26, 27] were propagated in MDCK cells.

### Construction of the NS1 and mutant expression plasmids

The NS1 expression plasmid (pEF-Bos-NS1) was constructed by cloning PCR-generated NS1 (PR8 strain) complementary DNA (cDNA) fragments into pEF-Bos [28] using engineered 5′ EcoRI and 3′ BamHI restriction sites. The NS1 R38A mutant expression plasmid was generated from the parental pEF-Bos-NS1 plasmid using KOD-plus site directed mutagenesis (Toyobo) according to the manufacturer’s instructions. Primer sequences are available upon request.

### Transfection and virus infection of cells

293 T RIG-I KO cells cultured in 6-well plates were washed once with phosphate-buffered saline (PBS) and inoculated for 10 min at 4 °C with multiplicity of infection of 0.01 (MOI = 0.01) of PR8-delNS1 virus suspended in PBS. To enable viral entry, the inoculum was replaced with DMEM containing 5% FCS and 100 U/ml penicillin and 100 μg/ml streptomycin at 37 °C and 5% CO_2_ for 1 h. Subsequently, infected cells were washed once with PBS and transfected with 1 μg of pEF-Bos empty vector or pEF-Bos-NS1 using the calcium phosphate precipitation method [29]. Five hours after transfection, transfection medium was replaced with DMEM containing 0.2% bovine serum albumin (BSA) and 0.5 μg/ml of 6-(1-tosylamido-2-phenyl) ethyl chloromethyl ketone (TPCK)-treated trypsin (Sigma Aldrich) at 37 °C and 5% CO_2_ for 48 h.

### Hemagglutination assay (HA assay)

Virus-containing supernatant was diluted 2-fold to generate serial dilutions. These serial dilutions were dispensed into a U-bottom 96-well plate before chicken red blood cells (RBC) (0.5%) was added. After a 30 mins incubation period at room temperature, the titer (expressed as HAU) were determined as the dilution of last well showing RBC agglutinating appearance (uniform reddish colour or halo shape) before the first well showing negative appearance (button shape or rolling RBC while tilting the plate).

### vRNA extraction and strand-specific RT-qPCR

Total RNA from cells was extracted as described previously [30]. Viral RNA (vRNA) from virus particles was extracted from supernatant of infected cells using the QIAmp viral RNA mini-kit (Qiagen) according to the manufacturer’s instructions. Strand-specific RT-qPCR was performed as described previously [31]. In brief, cDNA was generated from 5′ tagged primer targeting different segments of vRNA using Superscript III reverse transcriptase (Invitrogen). Two-fold diluted tagged-cDNA was subjected to qPCR analysis with Thunderbird SYBR qPCR Mix using a primer set targeting a segment-specific region. Quantification was performed on a StepOnePlus Real-Time PCR system (Applied Biosystems). Primer sequences are available upon request.

### Virus purification

293 T RIG-I KO cells were cultured in 10-cm dishes (Greiner Bio-One), infected with PR8-delNS1 virus at MOI 0.01 and transfected with 5 μg of pEF-Bos-NS1 using the calcium phosphate precipitation method. Forty-eight hours after infection and transfection, the supernatants were collected and centrifuged to remove cell debris. The cell-free supernatant was then treated with RNAse A at 5 μg/ml for 1 h at 37 °C to remove RNAs outside of virus particles. The RNAse A-treated supernatant was then layered on 30% (w/v) sucrose in a PBS cushion and virions were purified by ultracentrifugation at 141,000×g for 2 h at 4 °C using a CP100NX ultracentrifuge (Himac). The virus pellet was resuspended in PBS.

### Protein analysis by immunoblotting

Purified virus and infected cell lysates were subjected to SDS-PAGE. Proteins were electro-blotted onto polyvinylidene difluoride membranes (Millipore), which were then incubated with 5% skimmed milk at room temperature for 30 min followed by incubation with anti-NP, anti-M1 (#sc-69,824, Santa Cruz Biotechnology), anti-HA (#sc-52,025, Santa Cruz Biotechnology) and ant-NS1 (#sc-130,568, Santa Cruz Biotechnology) mouse monoclonal antibody (1:1000 dilution in TBST) overnight at 4 °C. After incubation for 1 h at room temperature with goat anti-mouse IgG conjugated with horseradish peroxidase (1:2000 in TBST) (Jackson ImmunoResearch), the blots were treated with Chemi-Lumi One Super (Nacalai Tesque) and the proteins were visualized using a LAS4000 (Fujifilm).

### vRNA-FISH and immunofluorescence

293 T RIG-I KO cells were grown on poly-D lysine-coated coverslips to 30% confluency, infected and incubated for the indicated time. Cells were fixed with 4% paraformaldehyde, followed by permeabilization with 0.5% Triton X-100 in PBS. Cells were then processed for RNA fluorescence in situ hybridization (RNA FISH) using the QuantiGene ViewRNA ISH Cell Assay Kit (Affymetrix) according to the manufacturer’s instructions. In brief, the cells were incubated with the segment 5 (Neg.) (#VF1–10583) probe set at a 1:100 dilution for 3 h at 40 °C, followed by pre-amplifier, amplifier and label probe (all at a 1:25 dilution) treatment for 30 min at 40 °C. After washing with PBS, the coverslips were used in an immunofluorescence assay. Coverslips were incubated in PBS/2% BSA overnight. Primary antibodies were diluted in PBS/2% BSA. Primary antibodies were either mouse monoclonal anti-M1 (1:200) (#sc-69,824, Santa Cruz Biotechnology) or anti-HA (1:200) (#sc-52,025, Santa Cruz Biotechnology) combined with rabbit polyclonal anti-NS1 (1:500) (#GTX125990) antibody. After 1-h incubation with primary antibodies at room temperature, coverslips were washed three times with PBS, then treated with Alexa Fluor 647 Donkey anti-Mouse IgG H + L (#A-31571, Invitrogen) and Alexa Fluor 488 donkey anti-rabbit IgG H + L (#A-21206, Invitrogen) supplemented with 1 μg/ml of DAPI to stain nuclei (all at a 1:1000 dilution in PBS/2% BSA) for 1 h at room temperature. After washing three times with PBS, cells were mounted with Fluromount-G (SouthernBiotech). Images were taken using the confocal laser scanning microscope TCS-SP8 (Leica Microsystems).

### IAV VLP production system

IAV VLPs were generated as described previously [25]. Briefly, sub-confluent monolayers of 293 T RIG-I KO cells in 6-well plates were transfected with 2 μg of the pCAGGS expression plasmid encoding M1, 1 μg of pCAGGS expression plasmid encoding HA, NA, NP, PB1, PB2 and NEP, 0.1 μg of pCAGGS expression plasmid encoding PA and M2, 1 μg of a firefly luciferase-encoding minigenome construct plus 0.75 μg of a Renilla luciferase construct (pRL-SV40) together with either 1 μg of pEF-Bos empty vector or 1 μg of pEF-Bos-NS1 using calcium phosphate precipitation method. As a negative control, PB2 was replaced by an additional PB1 plasmid. At 48 h after transfection, supernatants were collected and treated with 25 U/ml of Benzonase nuclease (Novagen) for 3 h at 37 °C to remove residual plasmids present in the supernatant. Nuclease-treated supernatants were then used for VLP infections of 293 T WT and RIG-I KO cells, which were pre-transfected with 1 μg of NP, PB1 and PB2, and 0.1 μg of PA. At 8 h after VLP infection, luciferase activities of donor cells (VLP-producing cells) and recipient cells (VLP-infected cells) were measured using the Dual-Luciferase Reporter System (Promega) with a Lumat LB 9507 luminometer (Berthod Technologies). Firefly luciferase activity was normalized to Renilla activity. Relative luciferase units were depicted as fold induction with respect to the mock control. Recipient cells were also subjected to immunofluorescence using mouse monoclonal anti-luciferase antibody (1:200) (#sc-74,548, Santa Cruz Biotechnology). For VLP purification, supernatant was layered on 20% (w/v) sucrose in PBS cushion and subjected to ultracentrifugation at 141,000×g for 2 h at 4 °C using a CP100NX ultracentrifuge (Himac). The VLP pellet was resuspended in PBS.

### Statistical analysis

Statistical analysis was performed using Prism 6 software (GraphPad). The Student’s two-tailed t-test was used to compare two groups (WT and other groups) and one-way ANOVA followed by Tukey’s test was used for multiple comparisons. *P* < 0.05 was considered significant (*P < 0.05, **P < 0.01).

## Results

### NS1 supports virus production in a manner independent of RIG-I and IFN-I antagonism

**Fig. 1:**
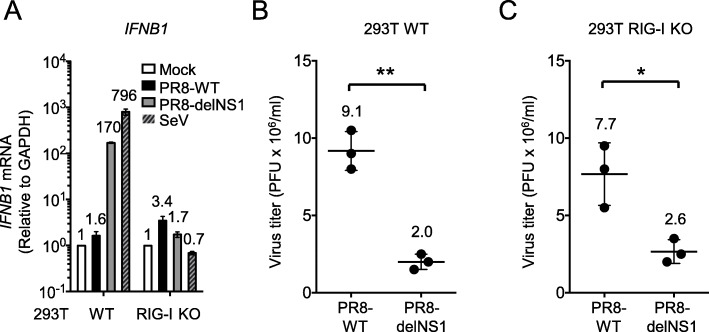
IAV replication in WT and RIG-I KO cells. **a** 293 T RIG-I KO cells were mock treated or infected with indicated viruses at MOI = 0.1. Twenty-four hours after infection, extracted total RNA was reverse-transcribed using random primers followed by qPCR with primers targeting *IFNB1.* WT 293 T (**b**) and RIG-I KO cells (**c**) were infected with PR8-WT or PR8-delNS1 at MOI 0.01. The virus yield was measured at 48 h post-infection by plaque assay using MDCK cells. The Student’s t test was used for statistical analysis (**P < 0.01, *P < 0.05). The data shown are the mean ± standard deviation from at least two independent experiments (*n* = 2 in (**a**), *n* = 3 in (**b**) & (**c**))

First, we explored the novel function of NS1 in supporting replication of IAV, independent of its RIG-I-antagonizing activity. To do this, we used RIG-I-deficient cells. *DDX58* KO HEK 293 T (293 T RIG-I KO) cells were infected with either PR8-IAV (WT or delNS1) or Sendai virus (SeV) and the expression of *IFNB1* mRNA was examined (Fig. 1a). As expected, *IFNB1* mRNA expression in PR8-WT infected cells was minimum. Although WT cells efficiently expressed the *IFNB1* gene upon infection by PR8-delNS1 and SeV, its induction in RIG-I KO cells was undetectable. IAV (PR8 strain) replication in WT cells was examined (Fig. 1b). As reported previously, PR8-delNS1 replicated significantly less efficient than PR8-WT virus [27] in Vero cell, which is an IFN deficient cell line. A similar result was obtained in RIG-I KO cells (Fig. 1c). Thus, PR8-WT is capable of replicating in RIG-I sufficient cells due to the RIG-I-antagonizing activity of NS1. In RIG-I deficient cells, PR8-delNS1 replicated significantly less efficient than PR8-WT, suggesting RIG-I-independent function of NS1 for viral replication.

**Fig. 2:**
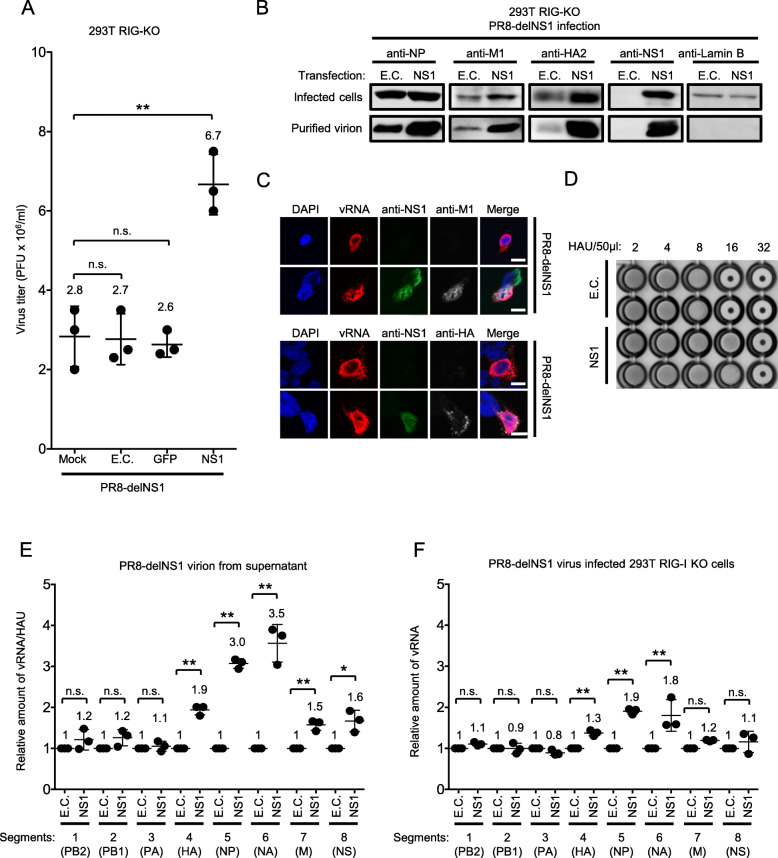
Effects of NS1 on virus yield. **a** 293 T RIG-I KO cells were infected with PR8-delNS1 at MOI 0.01 for 1 h and immediately mock transfected or transfected with empty plasmid control (E.C.) or those encoding GFP or NS1 protein. Virus yields were measured 48 h post-infection by plaque assay using MDCK cells. The data shown are the mean ± standard deviation from at least three independent experiments (*n* = 3). The Student’s t test was used for statistical analysis (n.s. not significant, **P < 0.01). **b** 293 T RIG-I KO cells were infected as in (**a**). Cells and culture supernatant were collected to prepare cell lysate and purified virion, respectively. Equal volumes of purified virus were subjected to immunoblot analysis using anti-NP, anti-M1, anti-HA and anti-lamin B antibodies. **c** 293 T RIG-I KO cells were infected with PR8-delNS1 at MOI 0.1 for 1 h and immediately transfected with NS1 expression plasmid or empty control plasmid. Cells were stained by fluorophore probe targeting segment 5 vRNA (vRNA, Red), anti- NS1 (green), anti-M1 (white), anti-HA (white) and DAPI (blue) 24 h after infection and transfection, and imaged by confocal microscopy. Scale bar, 10 μm. **d** Supernatant collected from (**a**) and virus quantity (HAU) was determined by HA assay using 0.5% chicken RBC. **e** vRNA was extracted from equal HAU from (**a**) and quantified by RT-qPCR. Levels of viral genome of virus containing supernatant obtained from E.C. transfected cells were set to 1. The Student’s t test was used for statistical analysis (*P < 0.05, **P < 0.01). The data shown are the mean ± standard deviation from at least three independent experiments (*n* = 3). **f** Cells in (**a**) were subjected to extraction for total RNA and vRNA was quantified by qPCR. Levels of viral genome obtained from E.C. transfected cells were set to 1. The Student’s t test was used for statistical analysis (n.s. not significant, *P < 0.05, **P < 0.01). The data shown are the mean ± standard deviation from at least three independent experiments (*n* = 3)

Although PR8-delNS1 is derived from PR8-WT and its genome sequence is expected to be identical except for the deletion in segment 8 [27], our sequence analysis revealed additional differences in the genome (data not shown). These differences may be due to spontaneous mutations and selection during passage. To confirm that the observed attenuation of PR8-delNS1 was due to the absence of NS1 and not intrinsic genome differences between the two viruses, we examined the effects of exogenous expression of NS1 on PR8-delNS1 virus infection in 293 T RIG-I KO cells (Fig. 2a). Ectopic expression of NS1, but not other control vectors or protein, rescued the attenuation of PR8-delNS1, suggesting that NS1 protein is required for efficient replication. Next, we examined PR8-delNS1 virion composition produced by RIG-I KO cells. Virion was purified from the culture supernatant of infected cells, either transfected with empty construct or expression vector for NS1 and subjected to immunoblotting (Fig. 2b). Total cell lysate was also prepared and analysed as a reference. As a result, M1 and HA2 (cleaved HA) were produced at higher levels in cells in complemented with NS1 (Fig. 2b, infected cells), confirming previous reports [18, 21]. In the virion, NS1, M1 and HA2 were detected at higher levels (Fig. 2b, purified virion). NS1 was also detected in the virion, confirming a previous report [22]. Expression of these proteins in cells were also confirmed by immunostaining (Fig. 2c). The high levels of viral proteins in virion fraction from NS1 expressing cells prompted us to examine the quantity of virus particle using HA assay. The amount of virus particle (expressed as HAU) produced in NS1 transfected RIG-I KO cells was approximately 2-fold higher than control transfected cells (Fig. 2d). For NP, the level in infected cells was comparable in the presence or absence of NS1 (infected cells, Fig. 2b) but virion fraction from cells complemented with NS1 contained a higher amount of NP (purified virion, Fig. 2b). The high level of NP in virion fraction is likely due to a higher number of virus particle contained in the supernatant. However, because NP is also a component of vRNP, it is possible that genome packaging is also affected in the presence of NS1. To test this hypothesis, genomic vRNA was quantified and normalized by HAU (Fig. 2e). Note that the quantification is not absolute copy number but relative ratio between the absence or presence of NS1. The result clearly showed that vRNA segments 4–8 were more efficiently packaged in the presence of NS1. To determine whether the increase of respective genomic vRNA in virion correlate to the its replication level in infected cells, we quantified vRNA in RIG-I KO cells. A comparable copy number of vRNA segments 1, 2, 3, 7 and 8 was observed irrespective of NS1 expression. However, significantly increased levels of vRNA segments 4, 5 and 6 were observed in NS1-expressing cells (Fig. 2f), suggesting that NS1 has a positive effect on vRNA copy number in a segment-specific manner. It is worth to note that packaging of segments 4–6 was more pronouncedly enhanced compared to enhanced accumulation of these segments in the infected cells, suggesting a role of NS1 in packaging.

### Effects of NS1 on viral genome expression, VLP production and genome packaging in the minigenome system

**Fig. 3:**
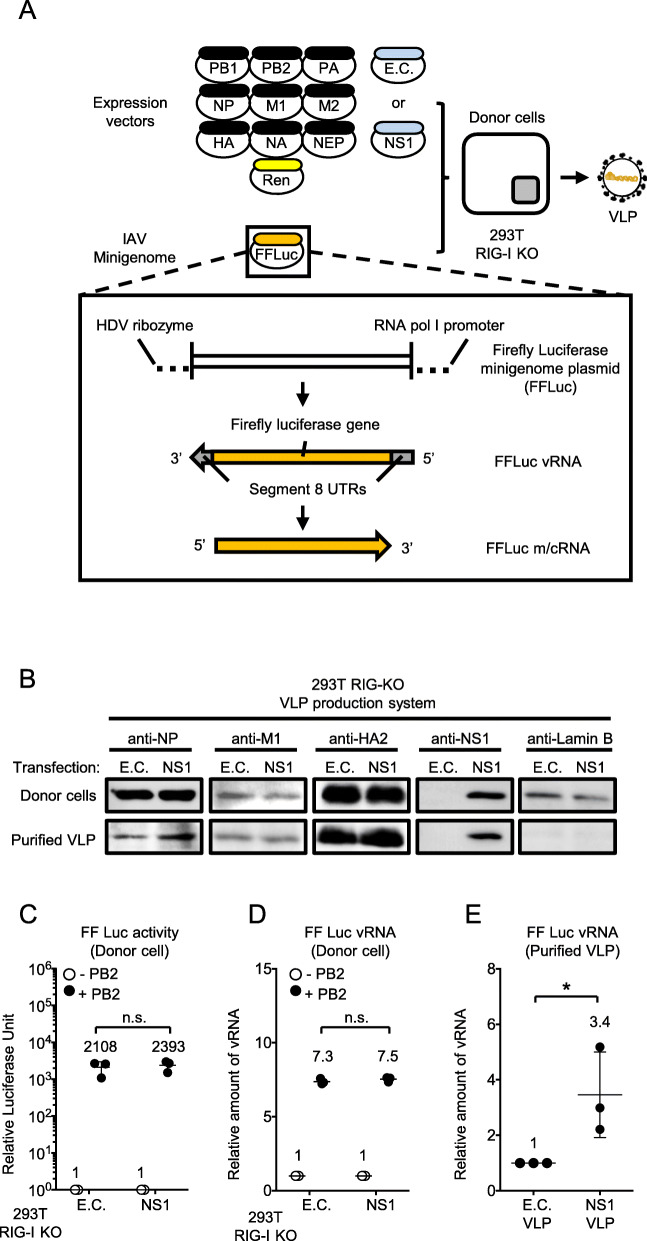
Effects of NS1 on viral replication in the VLP system. **a** 293 T RIG-I KO cells were co-transfected with eleven expression plasmids as described and a minigenome expression construct. Minigenome expression is regulated by RNA pol I as illustrated. **b** Twenty-four hours after transfection, cells and VLP released in the culture medium were harvested. VLP was further purified (Materials and Methods). Proteins in donor cells and purified VLP were detected by immunoblotting. **c** Luciferase activity in the donor cells was measured. Firefly luciferase activity was normalised by Renilla luciferase activity. Luciferase activity without PB1 expression was set to 1. The Student’s t test was used for statistical analysis (n.s. not significant). The data shown are the mean ± standard deviation from at least three independent experiments (*n* = 3). **d** Total RNA was extracted from the donor cells and minigenome was quantified by strand specific RT-qPCR. Levels of firefly luciferase vRNA obtained from cells without PB1 expression were set to 1. The Student’s t test was used for statistical analysis (n.s. not significant). The data shown are the mean ± standard deviation from at least three independent experiments (*n* = 3). **e** vRNA in the purified VLP was quantified by strand specific RT-qPCR. Levels of Firefly luciferase vRNA obtained from E.C. transfected cells were set to 1. The Student’s t test was used for statistical analysis (*P < 0.05). The data shown are the mean ± standard deviation from at least three independent experiments (*n* = 3)

The above results confirmed that NS1 increases IAV protein and some of the vRNA accumulation. To focus on packaging process more precisely, we next employed a virus-like particle (VLP) production system (Fig. 3a). 293 T RIG-I KO cells were co-transfected with plasmids, including those for expression of viral proteins, a Firefly luciferase reporter minigenome and a Renilla luciferase control reporter in the presence or absence of NS1 expression. First, viral protein expression in the donor cells and VLP was examined (Fig. 3b). Levels of viral proteins examined in the donor cells were unaffected by NS1 (donor cells, Fig. 3b). Likewise, the vRNA reporter copy number and luciferase expression in these cells were similar in the presence or absence of NS1 (Fig. 3c, d). In summary, viral protein and vRNA levels in donor cells were unaffected by NS1 expression. The levels of M1 and HA2 associated with VLP produced from donor cells were comparable in the presence or absence of NS1. However, there was a slight increase in the level of NP in VLP produced from donor cells expressing NS1 (purified VLP, Fig. 3b). NS1 was clearly incorporated in the VLP and its incorporation efficiency was comparable with that of the IAV virion (Fig. 2b and 3b). Interestingly, the minigenome copy number in the VLP significantly increased if NS1 was supplemented in the donor cells (Fig. 3e).

### Increased infectivity of VLP in the presence of NS1

**Fig. 4:**
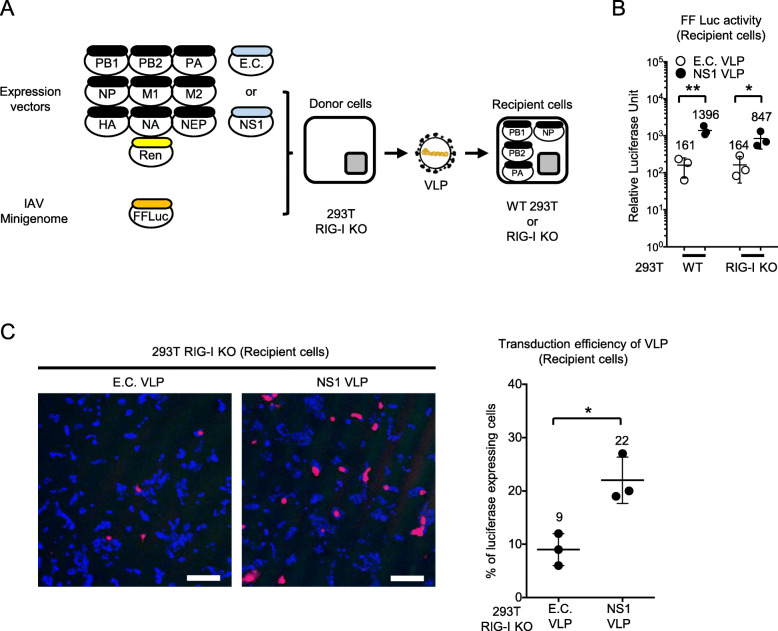
Effects of NS1 on gene transduction and infection by VLP in the recipient cells. **a** Schematic representation of VLP production and minigenome gene transduction and infection in the recipient cells. **b** VLP produced in the culture supernatant was transferred to the recipient cells derived either from WT 293 T or 293 T RIG-I KO cells. Luciferase activity was measured after 8 h. The Student’s t test was used for statistical analysis (*P < 0.05, **P < 0.01). The data shown are the mean ± standard deviation from at least three independent experiments (*n* = 3). **c** VLP-treated recipient cells (293 T RIG-I KO) were stained with anti-luciferase antibody (red) and DAPI (blue), and examined by confocal microscopy (left). Scale bar, 10 μm. Cells were quantified for luciferase expression (right). The Student’s t test was used for statistical analysis (*P < 0.05). The data shown are the mean ± standard deviation from at least three independent experiments (*n* = 3)

Next, we investigated the role of NS1 in the ability of VLP to transmit the minigenome to the recipient cells (Fig. 4a). VLP fractions produced from donor cells were inoculated to recipient cells. Similar levels of luciferase activity were obtained in WT and RIG-I KO cells infected with VLP produced in the absence of NS1 (Fig. 4b). In both recipient cells, significantly increased reporter activity was observed after infection with VLP produced in the presence of NS1. This was further confirmed by immunostaining of the infected cells (Fig. 4c).

### Involvement of the RNA-binding domain of NS1 in the increased viral infectivity

**Fig. 5:**
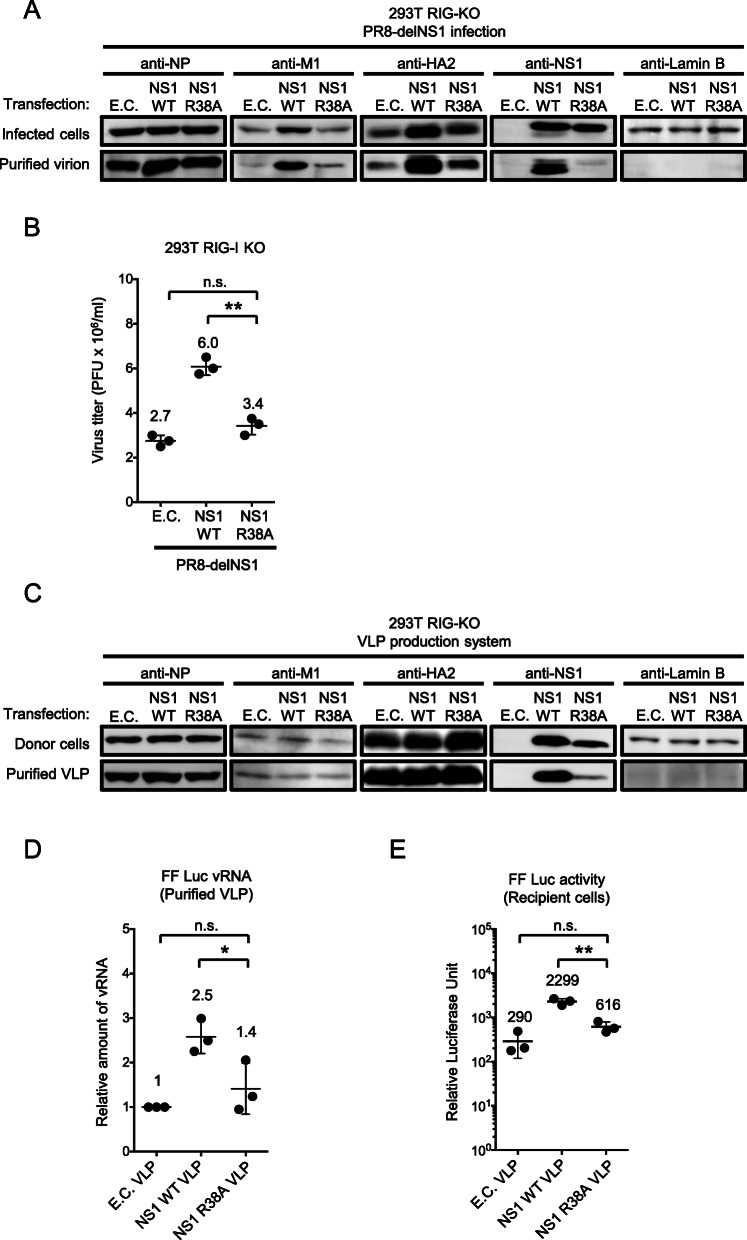
Involvement of RBD of NS1 in viral replication. **a** 293 T RIG-I KO cells were infected with PR8 delNS1 at MOI 0.01 and transfected with empty vector (E.C.), NS1 WT or NS1 R38A as described in Fig. 2a. Cell lysate and purified virion were analysed by immunoblotting. **b** Viruses released in the culture supernatant were quantified by plaque assay. The Student’s t test was used for statistical analysis (n.s. not significant, **P < 0.01). The data shown are the mean ± standard deviation from at least three independent experiments (*n* = 3). **c** 293 T RIG-I KO cells were used to produce VLP as described in Fig. 3a in the absence of NS1 or NS1 WT or NS1 R38A. Proteins in the donor cells or purified VLP were examined by immunoblotting. **d** vRNA extracted from VLP was quantified by RT-qPCR. Levels of Firefly luciferase vRNA obtained from E.C. transfected cells were set to 1. The Student’s t test was used for statistical analysis (n.s. not significant, *P < 0.05). The data shown are the mean ± standard deviation from at least three independent experiments (*n* = 3). **e** VLP was transferred to the recipient cells (293 T RIG-I KO, Fig. 4a) and luciferase activity was measured. The Student’s t test was used for statistical analysis (n.s. not significant, **P < 0.01). The data shown are the mean ± standard deviation from at least three independent experiments (*n* = 3)

We next investigated the involvement of the dsRNA-binding domain (RBD) of NS1. For this, we examined the R38A mutant of NS1. R38 located at helix 2 of the NS1 RBD and R38A mutation abolishes its dsRNA-binding ability [32, 33]. 293 T RIG-I KO cells were infected with PR8-delNS1 in the absence or presence of NS1 WT or NS1 R38A as in Fig. 2. Protein analysis of infected cells confirmed that expression of M1 and HA2 were increased by complementation of NS1 WT, as shown in Fig. 2b, but these effects were attenuated when complemented with NS1 R38A (infected cells, Fig. 5a). NS1 WT increased virion associated M1 and HA2, but NS1 R38A exhibited markedly reduced effects (purified virion, Fig. 5a). Similarly, NS1 R38A failed to fully rescue the attenuation of PR8-delNS1 (Fig. 5b). Incorporation of NS1 into the virion was also impaired by R38A mutation. We examined the effects of NS1 R38A mutation in the VLP system. 293 T RIG-I KO cells were used to produce VLP in the absence or presence NS1 WT or NS1 R38A (Fig. 5c). Immunoblotting analysis revealed that the control, NS1 WT and NS1 R38A-expressing donor cells had similar NP, HA and M1 protein levels (donor cells, Fig. 5c). The level of NP was slightly increased in VLP produced from donor cell expressing NS1 WT but not NS1 R38A (purified VLP, Fig. 5c). Of note, NS1 incorporation in VLP from NS1 R38A-expressing cells was significantly reduced (purified VLP, Fig. 5c). Similar results were observed in cell lysates (donor cells, Fig. 5c). Based on RT-qPCR, the amount of vRNA isolated from VLP produced from NS1 R38A-expressing donor cells was reduced by approximately 2-fold, comparable with empty control cells (Fig. 5d). We next asked whether these phenotypes affect VLP infectivity. VLP produced in the presence of NS1 WT or NS1 R38A was inoculated to recipient cells (293 T RIG-I KO cells) and luciferase activity was examined (Fig. 5e). VLP produced from NS1 R38A mutant-expressing donor cells exhibited significantly reduced luciferase activity.

## Discussion

In this report, we focused on the function of NS1 other than its antagonizing activity against innate immunity. By comparing WT and delNS1 viruses, in addition delNS1 virus complemented with ectopic expression of NS1 (Figs. 1 and 2), we found that NS1 significantly augmented the viral titer in RIG-I KO cells, which are practically defective in antiviral cytokine production (Fig. 1a). Our results strongly suggest that NS1 has multiple functions to assist in efficient viral replication. First, as reported previously [21, 34–37], NS1 increases viral protein expression in the infected cells, including M1 and HA2, but the expression of NP was unchanged, suggesting that the effects are segment-specific (Fig. 2b). Moreover, vRNA levels of segment 4, 5 and 6 were significantly increased by NS1 in the infected cells (Fig. 2f). However, the levels of vRNA and protein do not necessarily correlate, suggesting viral promoter-specific regulation of the synthesis of mRNA and vRNA, and translational regulation [18, 20, 21, 38, 39]. Thus, the mechanism of segment-specific viral gene expression by NS1 needs further investigation. The higher viral titer due to NS1 expression is well correlated with increased expression of viral protein and its amount in the supernatant (Fig. 2b), which results in enhancement of virus production (Fig. 2d). Also, genome (vRNA) packaging into the virion fraction is increased in a segment specific manner (Fig. 2e). We investigated the effects of NS1 using the VLP production system, where viral protein expression in the donor cells is independent of NS1 (Fig. 3b). NS1 significantly increased the transduction and infection efficiency of the VLP into the recipient cell (Fig. 4b, c). This was well correlated with the minigenome copy number in the VLP (Fig. 3e); however, the vRNA level in the donor cells was unchanged (Fig. 3d). Moreover, the amount of viral protein in VLP fraction was unaffected by NS1 (Fig. 3b). These results suggest that the second role of NS1 is to facilitate efficient genome packaging into the virion. As previously reported [22], we confirmed that NS1 is incorporated into the virion (Fig. 2b) and this incorporation was also observed in VLP (Fig. 3b). We explored the involvement of the dsRNA-binding ability of NS1 in IAV replication. R38A mutation impaired the incorporation of NS1 into the virion and VLP (Fig. 5a, c). This NS1 R38A was also unable to increase M1 and HA2 expression in infected cells (Fig. 5a) and thus impairs virus packaging and production, which leads to reduced infectivity (Fig. 5b). Moreover, NS1 R38A mutant impairs VLP genome packaging (Fig. 5d) and VLP-mediated luciferase transduction and infection (Fig. 5e). These results strongly support the hypothesis that the activities of NS1 for increasing viral protein expression and genome packaging are dsRNA binding-dependent. As each vRNA segment contains a dsRNA region formed by annealing 5′ and 3′ termini [40], this region may be a potential recognition site for NS1. The association of NS1 with vRNA may facilitate viral gene expression and genome packaging.

## Conclusion

NS1 has strong antagonizing activity against host immune responses, thereby masking additional functions in supporting viral replication. Our study revealed novel functions of NS1 in supporting efficient virus production and infectivity through enhancement of viral protein expression and genome packaging as one of the virus particle components. Our results suggest the RNA-binding function of NS1 could be a target for developing anti-IAV compounds.

## Data Availability

All data generated or analysed during this study are included in this published article.

## Abbreviations

cDNA: Complementary DNA
cRNA: Complementary RNA
E.C: Empty Control
FFLuc: Firefly Luciferase
GFP: Green Fluorescent Protein
IAV: Influenza A Virus
IFN: Interferon
mRNA: messenger RNA
NS1: Non-Structural protein 1
RBD: dsRNA Binding Domain
RNA pol I: RNA polymerase I
SeV: Sendai Virus
UTRs: Untranslated Regions
VLP: Virus-like Particle
vRNA: viral RNA
